# Targeting Age-Dependent Functional and Metabolic Decline of Human Skeletal Muscle: The Geroprotective Role of Exercise, Myokine IL-6, and Vitamin D

**DOI:** 10.3390/ijms21031010

**Published:** 2020-02-04

**Authors:** Clara Crescioli

**Affiliations:** Department of Movement, Human and Health Sciences, Section of Health Sciences, University of Rome “Foro Italico”, Piazza L. de Bosis 6, 00135 Rome, Italy; clara.crescioli@uniroma4.it; Tel./Fax: +39-06-36733395

**Keywords:** skeletal muscle, aging, metabolism, myokines, exercise, IL-6, vitamin D

## Abstract

In the elderly, whole-body health largely relies on healthy skeletal muscle, which controls body stability, locomotion, and metabolic homeostasis. Age-related skeletal muscle structural/functional deterioration is associated with a higher risk of severe comorbid conditions and poorer outcomes, demanding major socioeconomic costs. Thus, the need for efficient so-called geroprotective strategies to improve resilience and ensure a good quality of life in older subjects is urgent. Skeletal muscle senescence and metabolic dysregulation share common cellular/intracellular mechanisms, potentially representing targets for intervention to preserve muscle integrity. Many factors converge in aging, and multifaceted approaches have been proposed as interventions, although they have often been inconclusive. Physical exercise can counteract aging and metabolic deficits, not only in maintaining tissue mass, but also by preserving tissue secretory function. Indeed, skeletal muscle is currently considered a proper secretory organ controlling distant organ functions through immunoactive regulatory small peptides called myokines. This review provides a current perspective on the main biomolecular mechanisms underlying age-dependent and metabolic deterioration of skeletal muscle, herein discussed as a secretory organ, the functional integrity of which largely depends on exercise and myokine release. In particular, muscle-derived interleukin (IL)-6 is discussed as a nutrient-level biosensor. Overall, exercise and vitamin D are addressed as optimal geroprotective strategies in view of their multi-target effects.

## 1. Introduction

An undeniable consequence of the increase in human life expectancy that has occurred over recent decades is the compelling need for strategies to preserve human health and quality of life. Age extension over 65 years leads clinicians to deal with several comorbidities, such as metabolic and cardiometabolic disorders that entail important socioeconomic problems [1]. Since Homer’s representation of senescence, aging has been described as a process, classically named sarcopenia, closely associated with muscle decline and weakness [2]. The aging-related progressive loss of skeletal muscle mass, strength, and function correlates with severe adverse outcomes and physical frailty—a multidimensional syndrome clinically characterized by reduced reserves and resistance to stressors [3], immobility, disability, loss of independence, institutionalization, and mortality [4].

Skeletal muscle is the most abundant bodily tissue whereby the majority of postprandial glucose disposal occurs. Age-dependent skeletal muscle disruption and decline largely account for the development and exacerbation of whole-body metabolism disturbances, including insulin resistance (IR), impaired glucose tolerance, type 2 diabetes (T2D), dyslipidemia, hypertension, and central adiposity [5,6]. A hypothesis has been proposed that good health maintenance in later life relies on skeletal muscle healthy aging. Hence, interventions aimed at maintaining muscle homeostasis or targeting tissue senescence have developed along with challenges to their clinical translation [1].

Muscular integrity and function are regulated by several biological and lifestyle-related variables, namely, turnover and trophic factors, hormones, drug intake, smoking, and sedentary behavior, which are classified into muscular, and non-muscular factors, respectively [7]. Overall, physical exercise and vitamin D/vitamin D receptor (VDR) have emerged as powerful counter-measures against age-related muscular decline, not only through mass remodeling but, especially, by regulating muscle bio-secretory activity. Nowadays, healthy skeletal muscle is considered a proper secretory organ that can release, upon contraction, trophic and immunoactive regulatory factors called myokines. Through myokine release, muscle can regulate other organs’ metabolism and functions via autocrine, paracrine, and endocrine approaches [8]. Noticeably, VDR agonists and physical exercise can regulate some metabolic processes at the cellular level, acting on myokines, on the one hand, and counteracting altered energetic balance and IR in senescence progression, on the other. In addition, both exercise- and vitamin-D-induced cell signaling converge against inflammation.

This paper aims to offer an overview of cellular and molecular processes mediating aging and related dysmetabolism in skeletal muscle, herein referred to as an immune–endocrine secretory organ. In particular, muscle-derived IL-6 is discussed as a nutrient-level biosensor. In this context, physical exercise and vitamin D are addressed as useful strategies for geroprotection against age- and dysmetabolism-related harmful consequences.

## 2. Biomolecular Mechanisms of Age-Dependent Muscle Failure

Skeletal muscle aging is a multi-factorial condition based on the interactions of distinct dysfunctional systems and detrimental processes, each potentially representing a target for interventions. Even though the underlying mechanisms are quite far from being completely understood, age-related muscle loss encompasses several bioactivities, such as mitochondrial dysfunction, oxidative stress, inflammatory processes, metabolic and protein homeostasis perturbation, and senescent cell accumulation, which all converge in protein breakdown and tissue atrophy [9,10,11,12]. 

Aging seems to trigger “differential” resistance to I, with regard to glucose, protein, and lipid metabolism. Some older subjects can maintain sensitivity to I concerning glucose, but not protein synthesis. Indeed, age-dependent reduction of peripheral glucose utilization often occurs along the so-called “anabolic resistance” to I, a process, which seems to precede clinical manifestations, due to an unbalance in protein synthesis and degradation (proteostasis loss) [13,14,15,16,17].

The I-dependent cascade, including phosphatidylinositol 3-kinase (PI3K), protein kinase B/AKT, mammalian target of rapamycin (mTOR), and p70S kinase, is known to mediate both, IR and mass loss, likely due to the interconnection existing between glucose, protein, and lipid metabolism [18]. 

Restoring PI3K and AKT/mTOR function (i.e., with I-sensitizing drugs such as peroxisome proliferator-activated receptor (PPAR)γ agonists) results in a positive impact on both metabolism and muscle size [18]. In aging humans, progressive muscle loss has been mechanistically linked with visceral adiposity expansion and increasing intermuscular fat presence [18,19,20,21,22]. Intramyocellular lipid storage, mainly ceramide and diacylglycerol [23], can directly damage the I-dependent signaling cascade, impairing protein synthesis and glucose metabolism. The overexpression of muscle-specific PPARγ coactivator 1-α (PGC1-α) can counteract IR and reverse lipid-induced mitochondrial inefficiency through liver β-oxidation enhancement [24].

Muscle aging is also characterized by a pronounced reduction of mitochondrial activity and glycolytic type II fibers [25,26], both processes are strictly linked with IR and whole-body altered glucose metabolism. Besides the energy supply through adenosine triphosphate (ATP) necessary for contraction, mitochondria integrity ensures cell quality control and redox regulation, which are essential for skeletal muscle cell viability. An abnormal intracellular accumulation of reactive oxygen species (ROS) cannot be eliminated by the antioxidant defense system and ends in cell death due to ROS-induced biomolecular injuries in lipids, proteins, and DNA. ROS increase, as found in myosatellite cells of older subjects, is likely responsible for reduced regenerative potential [27]. Overall, an altered redox status impairs several biocellular/biomolecular processes, including calcium transport, cell differentiation, and autophagy [28], significantly contributing to muscle failure. This has been known since 1980, when Miquel et al. proposed “the mitochondrial free radical theory of aging” as the cause of ineffective antioxidant cellular defense due to mitochondrial DNA mutation and compromised electron transport chain [29]. In turn, mitochondrial mutations can further increase ROS production and accumulation, thus establishing a vicious loop, as confirmed in animals and humans [30]. To date, the mitochondrial free radical theory of aging seems somehow controversial, since sources other than mitochondria generate ROS [31,32,33]. Nevertheless, the pivotal role of ROS and mitochondrial dysfunction in muscle failure remains undeniable. 

In elderly humans, the release of damaged mitochondria in the extracellular matrix associates with plasma increase of proinflammatory cytokines [34]. Their interplay with ROS, altered mitochondria, and impaired cellular paths drives the orchestration of age-related injury in muscle and amplifies inflammation at extra- and intra-cellular levels.

In this scenario, inflammation and aging interact so tightly, mutually, and continuously so that they can be considered indistinguishable, merging into a single process named “inflammaging”.

### Inflammaging, Gerokines, and Antiaging Approach

The term inflammaging, first coined by Franceschi in 2000 [35], refers to low-grade chronic inflammatory status, which is generally observed in older populations and associated with increased circulating pro-inflammatory factors, proteases, cytokines, and chemokines (*chemoattractive cytokines*) (i.e., tumor necrosis factor (TNF)α; IL-1α/β; IL-6; IL-8; interferon (IFN)γ [36,37]; and their soluble receptors (R) IL-1Ra, TNFαsR, and IL-6sR). Some authors refer to these molecules as “gerokines”, constituting the “aging secretome”: The higher it is, the faster that inflammaging progresses [35,37,38]. Unlike C-reactive protein (CRP), considered a marker rather than a causative agent of inflammaging [39], proinflammatory cytokines/chemokines trigger and sustain inflammation at molecular and cellular levels. Some gerokines, such as TNFα and IL-6, are capable of modulating oncogenesis phases, so that their control might be particularly relevant [40,41]. Many different mechanisms converge in inflammaging, namely, immune cell dysregulation, genetic susceptibility, changes to microbiota composition and gut permeability, central obesity, cellular senescence, and inflammasome complex activation. The causes, effects, and mechanisms underlying inflammaging, now known as the common link of several diseases, have been thoroughly described elsewhere [39,42]. 

Concerning skeletal muscle, inflammaging and gerokines associate with tissue changes and deficits, leading to functional impairment [43]. Studies on more than 3000 men and women (aged 70-79 years) document a significant reduction in skeletal muscle area, mass, and strength in association with IL-6, IL-1, and TNFα/TNFαsR higher blood levels [44,45,46]. 

Presently, it is far from clear how inflammaging translates into skeletal muscle functional decline. However, TNFα and IL-6 seem to be key molecules since they drive the direct catabolic effect in myoblasts through inhibition of myogenesis and protein synthesis. In addition, the interaction with oxidative stress further unbalances protein synthesis and degradation equilibrium in favor of the latter [36]. The increase in protein breakdown and the consistent reduction in the number and size of muscle fibers—especially type II fibers, following TNFα-induced apoptosis—likely translate into functional and mechanical muscle impairment [47]. Along with muscle decrease, TNFα and IL-1β trigger fat mass increase and, in turn, proinflammatory adipokines can further accelerate muscle wasting to an extreme condition called sarcopenic obesity [48].

Among the inflammaging processes within skeletal muscle cells, IL-6- and TNFα-induced nuclear factor kB (NF-kB) activation play a major role in interfering with proteolytic cascade paths via the ubiquitin–proteasome system (UPS) [49,50]. The UPS catalytic part (20S proteasome), which is normally dedicated to degraded or misfolded protein removal, causes muscle wasting when chronically challenged by inflammation [51,52]. NF-kB over-activation hampers the muscular anabolic signaling cascade and leads to IR, likely wasting Akt and S6K1 signaling. Interestingly, NF-kB overexpression is present in the skeletal muscle tissues of older subjects with reduced muscular protein synthesis in response to feeding [13,53]. In this scenario, it is quite clear that IL-6, TNFα, or other gerokines, as well as the crosstalk between the TNFα/NF-kB/UPS cascade/Akt signaling, represent important targets to counteract skeletal muscle inflammaging.

As from in vivo and in vitro data, targeting IL-6 with cyclo-oxygenase inhibitors aids skeletal muscle mass and strength [54], while supplementation with proteins, antioxidants, and vitamins helps mitochondrial function and preserves antioxidant defense, also reducing ROS in diabetic experimental models [55,56].

In humans, specific studies on skeletal muscle inflammaging are still in their infancy. Some evidence exists in favor of protein supplementation, especially in T2D; obesity; menopause-related overweight; and in the context of eccentric or unaccustomed exercise [36], which evokes an immunoinflammatory-like response from muscular fiber microtrauma [57].

However, protein supplementation reduces creatine kinase but leaves unaltered circulating TNFα, IL-6, or CRP both in overweight subjects and well-trained populations [58,59,60,61]. 

Antioxidant vitamins C or E may help muscle mass and function after resistance training, even if inconsistent data have been reported, depending on the study participants’ age and training duration [62,63]. Vitamin K can suppress NF-kB signal transduction and ROS generation, likely through direct effects on muscle tissue. Older subjects with lower plasma vitamin K seem to retain higher mobility limitation and disability [64,65]. Vitamin D, classically classified as a steroid hormone, is well recognized to be a critical regulator for muscle function during all life spans, from intrauterine to late-phase life, due to anti-inflammatory, metabolic, and immunoregulatory actions, even though its antioxidant effect has been recently debated [66,67,68,69,70,71,72]. The wide-ranging effects of vitamin D on muscle function are addressed later in this review.

The use of antioxidants and anti-inflammatory substances in older populations is recommended with some caution, as interfering with ROS in muscle tissue might counteract exercise adaptation. Indeed, antioxidant supplementation can prevent mitochondrial biogenesis, I sensitivity, or mass gain by reducing ERK1/2, p38 MAPK, and p70S6 kinase phosphorylation [73,74]. Therefore, antioxidants may potentially be harmful to the elderly due to attenuation of training-induced anti-inflammatory effects.

## 3. Physical Exercise and Geroprotection: Maintaining the Secretory Function of Skeletal Muscle 

Physical exercise, after an initial inflammatory-like response, induces advantageous adaptations, essentially promoting mitochondrial biogenesis, re-establishing metabolic homeostasis, and counteracting detrimental inflammatory mediators [75,76]. This effect is important in the general population, but assumes particular significance in older subjects for fighting muscle failure [1,77]. Some studies distinguish between endurance exercise (low-resistance aerobic fitness at various intensities), acting on muscular oxidative capacity, and are reflected in better skeletal muscle and cardiovascular function, as well as resistance exercise (high intensity/resistance activity), acting on muscular mass and strength [78,79]. The high variability in outcomes due to the heterogeneity of exercise protocols and differences in participants (i.e., gender, age, physical status, and ethnicity), study extent, and physical performance measures makes it quite difficult to meaningfully assess the data [80]. Nevertheless, the positive impact of physical activity (mild/moderate/vigorous) and exercise vs. sedentary behavior is undeniably recognized. Indeed, skeletal muscle inactivity produces the same defects as aging on glucose/lipid-metabolism-dedicated intracellular machinery [81,82,83] and similarly increases the risk of T2D or metabolic syndrome [84]. Conversely, any type of physical activity reduces this risk in subjects aged 70 years or older [85]. In particular, aerobic exercise programs induce mitochondrial remodeling in the skeletal muscle of T2D patients [86], counteract ROS production, and reduce anabolic resistance by restoring, at least in part, the AKT/mTOR intracellular cascade [19,28,87].

Thus far, physical activity can be considered a non-muscle-specific geroprotector, in view of the induction/activation of biomechanisms delaying/counteracting cellular senescence, apoptosis, autophagy, and inflammation. Even more, skeletal myocytes upon contraction can release myokines, a set of small peptides closely connected with local muscular processes and other distant systems (endocrine, immune, and nervous), where they exert functional control [8,88].

### The Myokine Concept

Myokines have been recently proposed as frailty biomarkers because they are closely related to a person’s health status and biological age, which mirrors body age based on some biological indices (i.e., cytokines, DNA methylation, and physical activity) [10].

Aging, T2D, or inactivity triggers similar alterations in myokine signaling paths or levels [89]. Physical-inactivity-induced changes in the myokinome profile affect the transcription of about 4000 genes and promote the development of chronic diseases (i.e., cardiovascular diseases, neurodegeneration, and cancer). Rediscovering skeletal muscle as a secretory organ, which can produce immunoactive factors, generated the so-called “myokine concept”: The myokines released during physical exercise are fine-tuning mediators within the organ/tissue communication network [8,90].

This concept relies on the integration between contracting/working muscle and distinct/distal organ functions with bidirectional crosstalk, as recently proposed in [91]. Thus, working/contracting skeletal muscle has been reconsidered as a proper endocrine organ which, through myokines, exerts powerful anti-inflammatory and prometabolic effects [91]. In addition, some myokines can exert local effects via autocrine/paracrine mechanisms. 

In this scenario, a new piece is added to the puzzle of the beneficial consequences of exercise, which no longer solely depends on the reduction of adipose tissue and inflammation.

In 1961, Goldstein hypothesized a “muscle humoral factor” released into circulation during exercise as a result of muscle fiber damage [92]. With progress in this research field, myokines were identified as damage-independent signaling peptides with pluripotent beneficial effects [88,93,94]. Nowadays, the human secretory myokinome comprises hundreds of factors [95,96], even though just 5% of the myokinome has been ascribed to specific functions [91]. In addition, the myokine signature is emerging to be fiber-type specific and its expression/regulation might depend on exercise type, protocol, and duration (i.e., training vs. acute exercise) [96]. Nevertheless, the exquisite metabolic role of certain myokines, acting either locally or distantly, to regulate liver, adipose, or pancreatic tissue is undeniable. Robust evidence is limited to IL-6, IL-8, IL-10, IL-13, IL-15, IL-18, musclin, irisin, fibroblast growth factor (FGF)-21, myostatin, myonectin, and apelin. Those myokines, once dysregulated (i.e., as a consequence of sedentary behavior), contribute to IR, obesity, T2D, and muscle wasting. A detailed description here of each myokine function would be excessive and has been exhaustively reported elsewhere [89]. Herein, the focus is on IL-6, the prototypical metabolic myokine first discovered, which is significantly involved in energy balance and aging processes. 

## 4. The Metabolic Identity of the Myokine IL-6: An Energy Biosensor 

The specific field of myokines remained unexplored for several decades since the first observation of the “humoral factor” released in the blood upon muscular contraction [92], then identified as IL-6. In the 1990s, researchers ascribed the blood IL-6 exponential increase post-exercise (up to 75-fold, similar to post-traumatic condition) to neutrophils/macrophage tissue infiltration following muscular fiber disruption [97,98]. This hypothesis, indeed, is in line with the inflammatory facet of IL-6 released by immune system cells or adipocytes.

Conversely, later investigations identified human skeletal muscle cells, not immune cells, as the source of IL-6 released into the bloodstream upon muscular contraction within a framed temporal window after exercise [99]. Nowadays, it is well accepted that muscle-derived circulating IL-6 acts like a hormone and signals to the liver to start glycogenolysis and release glucose following the exhaustion of muscular glycogen deposits during exercise [99,100]. Those findings in humans were achieved in a series of elegant experiments documenting, during exercise, that the depletion of muscular glycogen content triggers IL-6 release; in this experimental condition, glucose intake attenuates or suppresses IL-6 release by the skeletal muscle [75,99,101,102]. Furthermore, the results obtained using arterial–femoral-venous differences, in order to compare resting versus exercising legs of the same subject, confirmed that only the exercising limb can release IL-6 [99,103].

Although, muscular IL-6 could derive from different cell types present in the tissue (i.e., fibroblasts, endothelial, and smooth muscle cells), skeletal myocytes have been documented to produce IL-6 [104,105]. In line with these results, we have reported that isolated human skeletal muscle cells, challenged by nutrient restriction to mimic a post-exercise condition, time-dependently release IL-6 [106], likely through post-transcriptional regulation, emptying intracellular IL-6 storage (personal communication). 

Thus far, the revolutionary concept of IL-6 “dual identity” has been proposed: A classical inflammatory cytokine, which is derived from immune cells or adipocytes, and participates in immune response to injury, or a myokine rediscovered as a fine-tuned metabolic biosensor, released upon skeletal muscle contraction. Intriguingly, this myokine is able to sense nutrient availability and initiate adaptive response, in order to maintain glucose balance and avoid muscular fatigue. In addition, muscular IL-6 has been shown to counteract TNFα, produced by adipose tissue and immune inflammatory cells, thereby, playing a pivotal role against IR and atherogenesis development [103]. 

In this scenario, it is fully conceivable how inactivity-triggered alterations of the myokine secretome, particularly IL-6, associate with higher risk of metabolic and cardiovascular diseases. Further roles of the myokine IL-6 seem to be sex-dependent, as suggested in a study on energy homeostasis in male and female mice [107]. 

As a result of this evidence, the term “myokine” has been used, not only to specify the source from which a molecule derives, but to identify a signaling peptide as a functional mediator of pluripotent beneficial effects [88,93,94,108]. 

The separation of the inflammatory and anti-inflammatory/metabolic effects of cytokines relies on the activation of the specific downstream intracellular cascade. The dual nature of IL-6 action involves specific critical mediators, such as NF-kB to promote inflammation, and fibrosis or AMPK to promote metabolic homeostasis [109]. This seems quite remarkable considering that AMPK-dependent intracellular signaling decline is characteristic of aging skeletal muscle and, at least in part, responsible for the decreasing adaptation to exercise, as compared with younger muscle. Exercise and related myokines can restore some metabolic deficiencies and ameliorate the sensitivity of signal transduction machinery through the reactivation of biomolecular regulators of mitochondrial plasticity (i.e., PGC-1, mTOR, and protein 53) [110,111]. In other words, it can be stated that myokines induce a metabolic virtuous circle.

We must emphasize that exercise alone is not enough to provide good muscle aging and optimal metabolic control, since a combination of several factors is necessary to obtain substantial pro-longevity benefits. Consistently, skeletal muscle decline is not defined as a univocal, independent disease. In view of the multidimensional nature of muscle failure, some characterizing factors have been recently separated into “muscle-specific” and “non-muscle-specific” biomarkers. While, the former are substantially linked to muscle mass turnover and trophism (i.e., procollagen type III N-terminal peptide, collagen type IV, 3-methylhistidine, and muscle-specific isoform of troponin T), the latter ones are linked to inflammation, lifestyle, nutrition, and hormonal control, as exhaustively reported elsewhere [7]. Notably, all factor interactions might be orchestrated at a central level by brain-associated molecules. This intriguing perspective importantly contributes to overcoming the old and inadequate paradigm of muscle wasting as a stand-alone disease. 

Herein, we discuss the importance of VDR agonists as important non-muscle-specific regulators of the metabolic machinery in myocytes.

## 5. Vitamin D and Skeletal Muscle Metabolic Integrity: Interplay with IL-6

There is a renewed interest in vitamin D’s beneficial effects on muscle integrity and metabolic balance, besides its classical functions in bone homeostasis.

It has been known for some time that VDR knockout mice show impairments in motor activity, muscle mass, and size and are prone to develop IR [112,113,114,115].

In humans, epidemiological studies have confirmed the central role of vitamin D in the maintenance of I sensitivity and muscular integrity. Metabolic and muscular defects in contraction, plasticity, and energy handling are associated with high prevalence of hypovitaminosis D in the general population, older or institutionalized subjects, and athletes as well [116,117]. Furthermore, fiber size reduction and type II fiber atrophy are found to be associated with low vitamin D levels. Hence, vitamin D supplementation became the clinical approach to overcome this hormone deficiency/insufficiency. To date, intervention studies report some contradictory data on the ability of supplementation to affect IR, muscle fiber size, strength, or power, which is likely due to inaccuracies in study power or subject selection [18,118]. 

Nevertheless, this molecule undeniably exerts multifaceted protective effects on mitochondrial gene expression and oxidative function due to the neutralization of oxidative stress and ROS generation, even if those processes are reported to be cell target-dependent [119]. Nowadays, the regulatory role of vitamin D in skeletal muscle function, strength, and recovery has been fully recognized [120]. Indeed, this hormone is shown to counteract IR and aging/metabolic impairments, improving glycemic control at the myocellular level. In mice, vitamin D can restore the efficiency of high-fat-diet-reduced I signaling and revert myosteatosis, tempering some mechanisms altered by high blood glucose, namely, the accumulation of advanced glycation end products (AGE), expression of AGE receptor (RAGE), and activation of NF-kB [121]. All those effects are mediated by non-genomic mechanisms of vitamin D.

In line with these data, we have shown in human skeletal muscle cells rapid I-like effects of the non-hypercalcemic VDR agonist elocalcitol, which modulates energy-dedicated intracellular machinery. In fact, elocalcitol, in addition to *GLUT4* gene expression upregulation, promotes GLUT4 intracellular trafficking in membrane lipid rafts (platform areas for GLUT4 internalization) and activates the I-dependent metabolic intracellular cascade, phosphorylating AKT, mTOR, ERK, and 4P-BP1 [106,122]. 

Notably, all those effects were found in association with an elocalcitol-dependent increase of IL-6 release induced by nutrient restriction, suggesting the existence of vitamin D/IL-6 crosstalk. 

Indeed, besides direct actions on skeletal muscle cell metabolism, vitamin D seems to exert beneficial effects by its interplay with myokines, namely, through negative regulation of myostatin, which works as a potent inhibitor of myokines.

Concerning the crosstalk with IL-6, some authors affirm that in aging skeletal muscle, the myokine is associated with VDR rather than with circulating vitamin D. This observation is based on a positive correlation found between intramuscular *IL-6* gene expression and VDR protein concentration in the older and mobility-limited population [123]. Although, the small sample size analyzed cannot provide definite mechanistic relationships, the authors indicate the possible involvement of intramuscular NF-kB. 

Based on this evidence and considering that VDR is inducible, important clinical implications of vitamin D supplementation in older/vulnerable subjects are likely conceivable. 

Besides vitamin D/myokine IL-6 crosstalk converging in muscular metabolic beneficial effects, another facet of the interplay between these two molecules should be considered. As previously addressed, long-lasting high systemic levels of IL-6 mirror inflammation and couple not only with general detrimental effects but, importantly, promote specific muscle degradation and atrophy. Those processes involve an increase in atrogene expression, such as Muscle RING-finger protein-1 (MuRF1) and atrogin-1, both upregulated in aged humans, along with their regulatory factors, including NF-kB [111,124]. Interestingly, atrogene downregulation following vitamin D treatment associates with IL-6 systemic suppression and results in significant improvement of muscle atrophy [125,126]. The mechanisms underlying this interplay are still to be fully elucidated; however, we can speculate that a pivotal role exists for the inhibition of NF-kB, a documented intracellular target of vitamin D [127]. Notably, NF-kB activation is prevented in VDR-agonist-treated human skeletal muscle cells [68].

In this scenario, further in vivo and in vitro investigations of possible IL-6/vitamin D synergy are recommended. Our ongoing research, while confirming IL-6/VDR agonist crosstalk, identifies glucose concentration as a critical condition for allowing the synergy to work (personal communication).

## 6. Conclusions

In conclusion, there is growing evidence on the importance of geroprotective interventions, which afford “good aging” of skeletal muscle tissue and, on the basis of clinical and basic research, is highly determinant of general health maintenance, especially in the elderly.

Muscular decline and metabolic diseases are tightly interconnected and share common biomolecular and cellular mechanisms, which represent potential intervention targets of the slow aging processes and ensure, as much as possible, good quality of life. Indeed, aging-dependent functional and metabolic muscle decline, when neglected, consequently leads to frailty and severe comorbid conditions. Although these concepts, based on scientifically documented data, are undoubtedly accepted, their translation into clinical practice is still far from being achieved. This limitation represents an important medical and socioeconomic urgency, since the debilitated elderly population is growing, resulting in considerable social expenses. Thus, higher accuracy in approaching these studies is a priority in order to avoid confounding and inconclusive data, which are found too often. Finally, we want to underline some limitations of the present review, namely, the lack of discussion of the pivotal role of signals from the muscle satellite cell niche or comments on gender- or sex-dependent variables. Nevertheless, we hope that reading this paper, while suggesting a major awareness of the resulting problems of the expansion of the older population, will help generate hypotheses that can improve the quality of life of the elderly.
